# Evaluation of autoantibody profiles in a cohort of new-onset pediatric systemic lupus erythematosus patients using autoantigen microarray technology

**DOI:** 10.1186/1546-0096-10-S1-A3

**Published:** 2012-07-13

**Authors:** Imelda M Balboni, Cindy Limb, Christy I Sandborg, Paul J Utz

**Affiliations:** 1 Stanford Medical Center, Stanford, CA, USA; 2 Stanford University, Stanford, CA, USA; 3 Stanford University School of Medicine, Stanford, CA, USA

## Purpose

Systemic lupus erythematosus (SLE) is characterized by the production of autoantibodies directed against many highly-conserved nuclear antigens. 15% of SLE patients develop disease in childhood or adolescence, and pediatric (pSLE) patients often have more severe disease onset and organ system involvement. Antigen microarray technology allows the comprehensive analysis of autoantibodies directed against hundreds of antigens with minimal amounts of sera. The purpose of this study was to characterize autoantibody profiles in a prospective cohort of new-onset pSLE patients at onset and within 6-9 months of disease onset and to determine if autoantibody profiles differentiate patients with distinct clinical manifestations such as nephritis.

## Methods

New-onset pediatric rheumatology patients meeting the revised ACR diagnostic criteria for SLE were eligible for this study. The study was approved by the Stanford University Institutional Review Board and informed consent was obtained prior to participation in the study. Sera from 51 pSLE patients and 17 healthy age- and sex-matched controls were evaluated using an 1128-feature antigen microarray manufactured with approximately 130 antigens. Demographic and clinical data from corresponding clinic visits were collected. Microarrays were probed with 1:200 dilutions of serum and a Cy5-conjugated goat-anti-human IgG secondary antibody, scanned with a GenePix 4000 scanner, and analyzed using GenePix 6.1 software to determine median fluorescence intensity minus background for each antigen. Significance Analysis of Microarrays (SAM) software was used to determine differences in autoantibody expression between pSLE patients and controls, within the pSLE group over time, and between pSLE patients with and without nephritis.

## Results

SAM identified increased reactivity against more than 50 autoantigens in new-onset pSLE patients compared to controls, with a false discovery rate (FDR) of less than 1%. Reactivity against many of these antigens decreased significantly by 6-9 months from disease onset. Subgroup analysis comparing patients with class III or IV Iupus nephritis to patients without significant nephritis demonstrated increased reactivity against several autoantigens including double stranded DNA, C1q and several types of collagen in patients with nephritis. Conversely, patients without significant nephritis had increased reactivity against other antigens including heat shock proteins and desmoglein.

## Conclusion

New-onset pSLE patients have a broad spectrum of antibodies directed against many autoantigens, including those not classically associated with SLE. In addition, preliminary subgroup analysis of pSLE patients with and without nephritis revealed distinct autoantibody profiles between the two groups. Further studies are currently underway to validate these findings and evaluate additional subgroups of patients with various clinical manifestations. Ultimately, autoantibody profiles may identify important biomarkers for more accurate diagnosis, prognostication, and treatment of pSLE patients.

## Disclosure

Imelda M. Balboni: None; Cindy Limb: None; Christy I. Sandborg: None; Paul J. Utz: None.

